# Electrophoresis of tear proteins as a new diagnostic tool for two high risk groups for dry eye: computer users and contact lens wearers

**Published:** 2011-08-25

**Authors:** A Chiva

**Affiliations:** Department of Clinical Chemistry, University Emergency Hospital Bucharest Romania

**Keywords:** Dry eye, computer users, contact lens, electrophoresis, tear proteins

## Abstract

Rationale: Dry eye is the most prevalent condition seen by the ophthalmologist, in particular in elderly. The identification of new common risk factors (computer use and contact lens wear) extends the disease among the young people. The early diagnosis of dry eye is essential, but difficult, because the biochemical changes in tear film usually occur before any detectable signs. Due its advantages, electrophoresis of tear proteins could be an important tool for diagnosis of tear film impairment in high risk groups for dry eye.

Objective: The role of tear proteins electrophoresis in early diagnosis of dry eye related to computer use and contact lens wear, as well as the biochemical changes in these high risk groups are presented.

Methods:  This review will summarize the actual data concerning the electrophoretic changes of tear proteins in computer users and contact lens wearers, two common high risk groups for dry eye.

Discussion: Electrophoresis of tear proteins using automated system Hyrys–Hydrasys SEBIA France is an important tool for early diagnosis of tear film alterations and monitoring of therapy. The quantification of many proteins in a single analysis using a small quantity of unconcentrated reflex tears is the main advantage of this technique. Electrophoresis of tear proteins should became a prerequisite, in particular for computer users less than 3h/day, as well as at prescribing contact lenses.

Abbreviations: DED– dry eye disease, EGF–epidermal growth factor, IL interleukins, MMP–metalloproteinase, ELISA– Enzyme–linked immunosorbent assay, SDS– sodium dodecyl sulfate, CVS– computer vision syndrome, CLRDE– contact lens– related dry eye

## Introduction

DED is the most common condition seen by the ophthalmologist worldwide. The prevalence varies between 7.4%  and  33.7%, depending on the study cited, the population surveyed and the diagnostic protocol [1]. DED always affects women more than men, in particular elderly [2–5]. The identification of new factors that can lead to DED (computer use, contact lens wear, office environment) extend the presence of disease among the young and make of it a real public health problem. 

The early diagnosis of DED is very important because the biochemical changes usually occur before the detectable clinical signs and symptoms. Without treatment, DED lead to serious complications with a significant impact on visual function and quality of life [1,6,7]. More of the standard tests currently used for DED diagnosis (Schirmer test, tear break–up time, fluorescein staining, meibometry) are not sensitive enough [8,9]. The identification of new biomarkers could be useful, but some difficulties in their measurement limit the use as routine analysis.

This review is aimed to present the role of tear proteins electrophoresis in early DED diagnosis, as well as biochemical changes of tear proteins in two of high risk groups for DED (computer users and contact lens wearers).

### Pathogenesis and risk factors for DED

DED is a multifactorial disease. The excessive evaporation and tear deficiency production are considered the main  mechanisms which can act individually or concomitantly [10,11].

In 2007, International Dry Eye Workshop (DEWS) improved the definition of DED by the inclusion of tear film hiperosmolarity and inflammation of the ocular surface in the development of disease [12,13]. In the same year, Baudouin published a new approach in the understanding of the pathogenesis of DED to explain why the disease occurs in some particular cases (contact lens wear, chronic allergy, or systemic or topical drugs) and why the long lasting effect remains even when the casual factors are removed [3]. This theory indicates two independent or complementary loops with tear film instability/hyposecretion as a key. In the first loop, tear film instability, neurogenic inflammation and apoptosis are closely linked. The second loop involves the eye lids and the lipids. Tear film instability is able to generate changes in bacterial flora in conjunctiva and eye lids leading to the release of endotoxins, lypopolysacharides and/or lipase activation and causing eyelid inflammation, meibomian gland dysfunction and lipidic changes [3].

In time, a plethora of risk factors have been identified. Many epidemiological studies highlight increasing age, menopausal and postmenopausal women, chronic androgen deficiency and oral contraceptive treatment as the most important factors that influence the tear secretion, meibomian gland function, and goblet cell density leading to DED [1]. In last years, DEWS and Delphy Panel add new factors in individual risk for DED, such as the environment (relative humidity, air conditioning, extreme temperature), use of systemic drugs or contact lens wear [14]. In addition, some social and dietary habits (smoking or alcohol consumption) could increase the incidence of DED [1].

The complexity of pathological mechanisms and the multitude of risk factors suggest that the development of new tear biomarkers became a necessity in order to an early and accurate diagnosis of DED.

### Tear biomarkers for DED

The early diagnosis of DED is essential, but difficult. The disease is often ignored; the visit to ophthalmologist being done only when symptoms become severe and when the biochemical changes at tear level have occurred. Many patients do not recognize the symptoms of DED or not report to the physicians. Thus, many cases remain under diagnosis and an impairment in visual function, work place productivity and quality of life appear [6,7].

In an ongoing effort to identify new specific tear biomarkers, the major event was the definition in 1998 of ‘lacrimal functional unit’ as an integrated system that includes not only lacrimal gland, but also ocular surface and interconnecting innervations [15]. Since then, a large variety of ocular investigations have been developed including both clinical assessment of ocular surface and tear film.

Tear meniscus measurement, meibometry, fluorophotometry, evaporation rate and thermography are the most useful investigation in DED. Impression cytology, a standard for ocular surface cells alteration, in conjunction with confocal microscopy, flow citometry, and molecular biology also play an important role [8].

Biochemical analysis of tear film brought about a significant improvement in diagnosis of DED, allowing the differential diagnostic of aqueous tear deficiency and evaporative dry eye [8], as well as the prediction of  the onset of more extensive clinical signs [16]. Many tear biomarkers may be tested using a wide variety of analytical procedure (electrophoresis, ELISA, high performance chromatography in thin layer, immunonephelometry). Tear osmolarity, electrophoresis, and measurement of major tear proteins (lactoferrin, lysozyme) remain the most useful analysis [8,17]. Additional tests may be performed to quantify inflammatory cytokines, matrix metalloproteinases, EGF, aquaporin 5, lipids and lipid peroxides for supplementary information about lacrimal gland dysfunction, the presence of a inflammatory reaction and oxidative stress, as well as altered distribution of tear lipid [8,17–20]. Decreased levels of EGF, a polypeptide produced by lacrimal gland that stimulate the growth of corneal epithelial cells and increased levels of aquaporin 5, an  integral membrane protein located in lacrimal gland and corneal epithelium that regulate the flow of water are strong evidences for DED [8,17]. Inflammatory (IL6, IL8) and proinflammatory cytokines (IL 1 alpha and beta), as well as matrix metalloproteinase MMP 9 are considered tear specific biomarkers for a local inflammatory reaction [18,19]. The lipid peroxides and myeloperoxidase activity could be additional tools in assessment of local oxidative stress, exacerbated by the inflammatory reaction and favored by the decreased level of lactoferrin and lysozyme, two antioxidant proteins [8]. Phospholipase A2, mucins and lipids can also be tested, but some difficulties in dosage method considerably limit their use as current tear biomarkers [20].

Despite the multitude of biomarkers and the great variety of analytical procedure, major limits still exist. The lack of standardization, the complexity of some analytical procedure that require specialized laboratories are considered the most important. The small quantity of tears that can be collected is another limit that can be removed using some stimulation procedure. Although chemical or mechanical stimulation could lead to an increased level of albumin originating from serum, the reflex tears can be used, especially in severe dry eye [8]. The results should be interpreted taking account the false increased albumin level. The use of filter paper [21] or the dilution of tears using saline solution and reconcentration [8] could also improve the sample collection, but the proteins absorption on filter paper and some difficulties in concentration technique are the main disadvantages.

### Electrophoresis of tear proteins as diagnostic tool for DED; a special focus on computer users and contact lens wearers, the high risk groups for DED

Electrohoretic analysis of tears protein is the key both in early diagnosis and prevention of DED, especially in groups with high risk of ocular complications associated with dry eye (computer users, contact lens wearers). 

Although SDS polyacrilamide gel electrophoresis provides good information about the major tear proteins, agarose gel electrophoresis using automated system Hyrys–Hydrasys SEBIA France considerably improves the resolution and the sensitivity of the test [22]. The main advantage remains the identification and relative quantification of many proteins in a single analysis. The interferences common to other types of electrophoresis (the human intervention in staining and destaining of electrophoregrams, the proteins absorption on filter paper or the less availability of concentration technique) are totally removed using SEBIA technique [8,22]. Only 5 micro l of reflex unconcentrated tears are necessary for test, collected in glass capillaries using a non invasive procedure.

About 15 proteins could be identified by SDS agarose gel, most of them having clinical value (lactoferrin, lysozyme, albumin, proteins 20–60 kDa, immunoglobulins) [22]. 

Lactoferrin (24–27% of total tear proteins), a multifunctional single chain polypeptide with anti–inflammatory, bacteriostatic and antioxidant properties and lysozyme (44–47%), a glycolytic enzyme with antimicrobial activity [16,23] are the most important peaks that can be detected on SEBIA electrophoregrams [22]. Produced by the acini of the main lacrimal gland, these proteins provide good information about a lacrimal gland dysfunction, representing an index of its function [16,17]. Moreover, decrease of their level is a good indicator for a inflammatory reaction, low antioxidant capacity, as well as predisposition for microbial infections (in particular lysozyme) [22,24]. Although electrophoresis, immunonephelometry and ELISA  are standard methods for lactoferrin and lysozyme measurement, the semiquantitative analysis continues to be used because their availability for the ophthalmologists [8]. 

Serum albumin can be also identified on SEBIA electrophoregrams [22]. As result of blood–occular barrier damage in conjunctival vessels, the increase of albumin level is detected, indicating an early exudation [16]. 

A heterogenous group of proteins with seric and lacrimal origin and molecular weight between 20 and 60 kDa can be detected as multiple bands, so called proteins 20–60 kDa [22]. Lipocalins, the major proteins of this group, provide good information about contact lens tolerance [20]. By contrast, immunoglobulins can be detected as single band, providing information about the presence of a local inflammation [8].

#### Computer users

For many years, computer is an important part of our everyday life. Intensively used, can lead to some ocular symptoms (ocular fatigue, irritation, redness, blurred or double vision, photophobia), collectively referred to CVS [25]. 

Evaporative dry eye is the most frequent condition associated with CVS. In the same time, a pre–existing DED may exacerbate CVS symptoms. The main cause of DED in CVS is reduced blinking [16]. Environmental factors (low humidity, indoor environment), as well as age and sex may also contribute to DED in CVS [1]. For these reasons, computer users require a careful monitoring as an important part of early diagnosis and prevention of complications related to DED. 

Tear proteins electrophoresis using automated system SEBIA France is the newest test for assessment of dry eye in CVS. It has been used in the Electrophoresis Laboratory of our hospital since 1998. No previous study about the use of this electrophoretic technique as well as about tear electrophoretic profiles at computer users has been published. 

**Figure 1:**
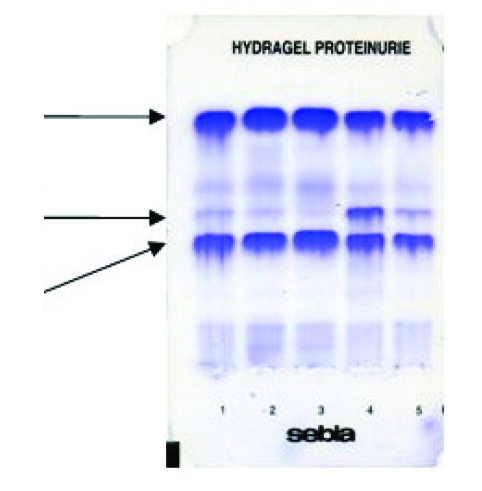
SDS agarose gel electrophoresis of tear proteins in computer users >3h/day (position no.1–2), <3h/day (position no.3) and in intolerant contact lens wearers (position no.4–5) before treatment. Lactoferrin, lysozyme and albumin as tear biomarkers for tear aqueous deficiency, inflammatory reaction, and contact lens intolerance are indicated by arrows

The decrease of lactoferrin and lysozyme levels, as well as the increase of albumin are the most common electrophoretic changes in dry eye associated with CVS. No variations or supplementary bands have been reported for proteins 20–60 kDa (Figure 1) [22]. The amplitude of these changes are correlated with time spent at the computer. The most expressive variation have shown lactoferrin and albumin in group >3h. The low levels of lactoferrin and lysozyme are strong evidences for a double etiology of DED in group >3h/day (excessive evaporation and tear aqueous deficiency) [22]. This findings confirm the theory in which more than one layer of tear film is often altered in DED [2]. Thus, lactoferrin and lysozyme, as main proteins of aqueous layer, can be useful markers for differential diagnosis of aqueous tear deficiency from the excessive evaporation. Moreover, the increased level of albumin in group > 3h demonstrated that a infraclinic inflammation has occurred and this tear biomarker, as well as lactoferrin could provide good information about the severity of this process [22].

**Figure 2:**
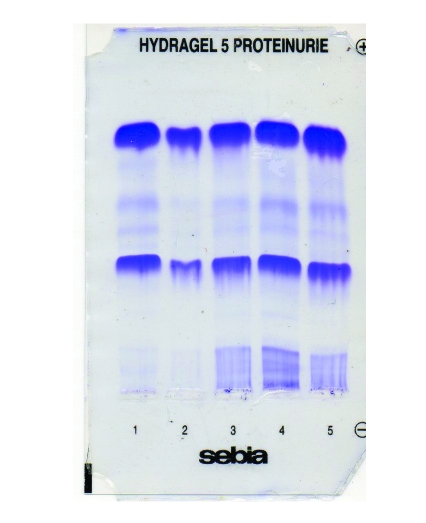
SDS agarose gel electrophoresis of tear proteins in computer users (position no.1–3) and contact lens wearers (position no.4–5) after treatment. The normalization of tear biomarkers values is good indicator for a successful treatment

No correlation between lactoferrin content, Schirmer test, and clinical signs in group <3h/day have been reported. This is a strong evidence that, in this group, biochemical changes occurs before clinically detectable symptoms and signs. In group>3 h/day, the electrophoretic changes has been correlated both with symptoms and Schirmer test. Moreover, lactoferrin levels below 18% are critical, leading to severe ocular surface disorders and requiring rapid therapy. The monitoring of lactoferrin level during three month of therapy with lubricant eye drops indicates a gradual return to normal values concomitant with reducing of dry eye discomfort (Figure 2)[22].

All these data suggest the great interest in use of tear protein electrophoresis as routine analysis for early detection of biochemical changes that can lead to tear film impairment, monitoring of therapy and assessment of complications risk (Table 1).

**Table 1:** Diagnostic and prognostic value of tear proteins assessed by agarose gel electrophoresis using automated system Hyrys–Hydrasys SEBIA

High risk groups for DED	Biochemical markers in tear film	Diagnostic and prognostic value
Computer users	Lactoferrin, lysozyme	Differential diagnosis of excessive evaporation (normal level) and tear aqueous deficiency (decreased level)
	Decreased lactoferrin level; Increased albumin level	Inflammatory reaction
	Decreased lactoferrin and lysozyme levels	Early diagnosis of DED
	Decreased level of lactoferrin <18%	Risk estimation for severe complications
	Normalized concentration of lactoferrin	Assessment of treatment efficacy
Contact lens wearers	Lactoferrin, lysozyme	Differential diagnosis of excessive evaporation (normal level) and tear aqueous deficiency (decreased level)
	Decreased lactoferrin level; Increased albumin level	Inflammatory reaction
	Decreased lysozyme level	High risk of infections
	Decreased lactoferrin and lysozyme levels; Increased albumin and lipocalins levels	Contact lens intolerance
	Normalized concentration of lactoferrin	Assessment of treatment efficacy

#### Contact lens wearers

CLRDE is the most common complication among the contact lens wearers. The frequency is about 50%, the women being between 1.5 and 2 times more likely to develop CLRDE as are men [4].

The tear film stability play an important role in contact lens tolerance. Although many potential mechanisms has been described, such as increased tear evaporation, inflammatory reaction, increased osmolarity, lens dehydratation, the etiology still remains unclear [26].In turn, contact lens alter the structure of tear film, as well as evaporation rate [27]. Thus, the presence of contact lens reduces the biochemical changes across the ocular surface. Moreover, contact lens may function as a barrier that reduce the amount of oxygen necessary for corneal function. As a result, a corneal hypoxia can cause an increase in the susceptibility of bacterial infection [26]. 

All these findings suggest that the early detection of changes in tear content, in particular in tear proteins, is essential in assessment of tear film structure and in prevention of ocular complications, as an important part of contact lens tolerance.

The tear film parameters that can be used as contact lens intolerance biomarkers are still controversial. Glasson et al. have reported that tear film stability, tear volume, and the symptoms are the best variables that predict the contact lens intolerance [27]. The same study revealed that the activity of secretory phospholipase A2, oxidized lipids level, and tear lipocalin concentration were significantly different between contact lens tolerant and intolerant wearers and correlated with the symptoms. The volume of aqueous tears that cover the ocular surface and the tear lactoferrin level are also related to DED in contact lens wearers. Lactoferrin can be a good predictor of tear film stability or volume because its level is associated with tear production. 

These findings are in good agreement of electrophoretic study of tear protein using SEBIA automated system. Thus, it has demonstrated that lactoferrin and lysozyme are the most important tear biomarkers for contact lens tolerance and a fall in lactoferrin content (below 18%) is associated with contact lens intolerance. A slightly decrease of tear lactoferrin and lysozyme levels were the most common electrophoretic changes (Figure 1) [22]. High levels of immunoglobulins have been reported in several studies, indicating an inflammatory as well as foreign body reactions [8,24]. In a good agreement with the study of Sariri et al., the SEBIA electrophoregrams have shown that in tolerance contact lens the lactoferrin seem to be not much affected by the use of lens [22,24]. In intolerance contact lens, the amplitude of electrophoretic changes is associated with time that the contact lens were weared and the severity of inflammatory reactions. A 18 month survey of contact lens wear has identified a gradually decrease of lactoferrin and lysozyme levels, as well as an increase of tear albumin, as a result of an inflammatory reaction. The difference of tear lactoferrin and lysozyme content between the first and the last month of study was about 10%. After therapy, a gradually return to normal values of lactoferrin and lysozyme with a concomitant  reduction of ocular discomfort have been reported (Figure 2)[22]. 

All these data suggest that electrophoresis of tear proteins is a useful test for assessment of contact lens intolerance. Because of its ability to measure lactoferrin, lysozyme and albumin levels in a single analysis, it can provide good information about the presence of tear aqueous deficiency and/or inflammatory reaction (Table 1). In the same time, since lysozyme is an antibacterial tear protein, low levels can indicate a poor antimicrobial defense capacity of tears and a high predisposition for ocular infections. Moreover, the monitoring of lactoferrin level is the best indicator for the efficacy of therapy [22]. For these reasons, electrophoresis of tear proteins should be included in the protocol for diagnosis and monitoring of CLRDE, as routine analysis, both at prescribing and during contact lens wearing.

## Discussions

DED is the most common complication among computer users and contact lens wearers. The under diagnosed cases, as well as the biochemical changes that can occurs before clinically detectable symptoms make the early diagnosis difficult, but essential.  

Although many biomarkers of tear film could be used, the difficulties in some analytical procedures that require specialized laboratories and the great amount of tears necessary for analysis are considered the major limits [8]. Mechanical stimulation of tearing or dilution in saline solution and reconcentration could be used, but the proteins absorbtion on filter paper and some difficulties in concentration procedure cannot be removed. However, the reflex tears can be used in severe dry eye [22,24]. 

Electrophoresis of tear proteins using SEBIA automated system is able to remove most of these limits. The relative quantification of many interesting proteins in a single analysis and the small quantity of unconcentrated reflex tears necessary for test are the main advantages that make of electrophoresis the most important tool in preliminary assessment of protein changes related to DED. Lactoferrin, lysozyme, proteins 20–60 kDa, albumin and immunoglobulins are the most important proteins that can be identified [22].

The decrease of lactoferrin and lysozyme as well as the increase of albumin are the most common electrophoretic changes in high risk groups for DED [22]. The amplitude of these changes has been correlated with the severity of disease. Low levels lactoferrin and lysozyme suggest an early impairment of lacrimal gland function before the changes of Schirmer test values [16] as well as a double etiology of ocular complications (excessive evaporation and tear aqueous deficiency). High level of serum albumin indicates an early exudative process in evaporative DED [16], making of it an important inflammatory marker [22]. 

Some tear biomarkers play a particular role in assessment of contact lens intolerance. Low level of lactoferrin and high level of lipocalins are best indices for lens intolerance [22,27]. In addition, the increase level of immunoglobulins could provide information about the response to foreign body [24]. Moreover, a decrease of lysozyme levels indicates a high predisposition to infections [22,24]. 

Tear proteins electrophoresis can be also used for monitoring of therapy, both in contact lens wearers and computer users. As it previously mentioned, lactoferrin levels below 18% is considered critical and require rapid therapy. A gradual return of its level during the therapy is the best indicator for its efficacy [26].

All these data suggest that tear proteins electrophoresis is an important tool for the early diagnosis of tear film impairment, monitoring of therapy and contact lens intolerance. Thus, electrophoresis should became a prerequisite, in particular for computer users who spent less than 3 hours a day as well as at prescribing and during contact lens wearing [22]. Despite the great variety of tear biomarkers, none of these has absolute clinical value. Their results should be correlated with other ocular surface investigations and symptoms for a high accuracy diagnosis.
